# Use of Lower-Leg Bathing to Facilitate Exercise Therapy in a Patient With Severe Pulmonary Arterial Hypertension: A Case Report

**DOI:** 10.7759/cureus.72732

**Published:** 2024-10-30

**Authors:** Yusuke Takahashi, Daido Miyamoto, Kakeru Hasegawa, Tomohito Suzuki, Hiroyuki Watanabe

**Affiliations:** 1 Department of Rehabilitation Medicine, Akita University Hospital, Akita, JPN; 2 Department of Cardiovascular Medicine, Akita University Graduate School of Medicine, Akita, JPN; 3 Department of Cardiology, Akita University Graduate School of Medicine, Akita, JPN

**Keywords:** case report, exercise therapy, heart failure, lower leg bathing, pulmonary arterial hypertension

## Abstract

Waon therapy (WT), which in Japanese means soothing warmth, is a repeated sauna therapy that improves cardiac and vascular endothelial function in patients with chronic heart failure (CHF). However, lower-leg baths can be easily performed anywhere. Here, we report a case in which leg baths were used to alleviate the symptoms of heart failure and enable the introduction of exercise therapy. A woman in her 70s with collagenous pulmonary arterial hypertension (class Ⅲ) associated with scleroderma was on home oxygen therapy; however, she experienced difficulty in performing active exercise therapy owing to shortness of breath on exertion and hypotension. As an alternative to WT, three sessions of lower-leg bathing at 42°C were performed weekly for three weeks. After lower-leg bathing, the patient’s peripheral blood flow improved, and her clinical symptoms were alleviated; as such, exercise therapy could be introduced. However, tricuspid regurgitation pressure gradient and N-terminal pro-B-type natriuretic peptide returned to pre-intervention levels nine months after discharge. Although lower-leg bathing can temporarily alleviate the patient’s symptoms, its effect is not long-lasting. Although lower-leg bathing has no long-term effects, it may be a treatment option for severe pulmonary hypertension (PH) when exercise therapy is difficult to introduce.

## Introduction

Pulmonary hypertension (PH) is a chronic progressive disease in which the pulmonary arterial pressure increases due to various diseases and related conditions, including chronic heart and lung diseases. Patients with PH often complain of dyspnea, fatigue, difficulty in performing activities of daily living, and low exercise tolerance [1]. Although the treatment of PH is advancing rapidly, thus far, side effects have been reported for all the associated specific medications. Most patients with PH remain symptomatic and have decreased mobility, quality of life, and survival despite receiving optimized medical therapy [2].

Exercise therapy is a non-pharmacological treatment for heart failure. The first randomized controlled trial of exercise therapy for PH was conducted by Mereles et al., who reported its safety and efficacy [2]. Cardiac rehabilitation, which mainly includes exercise therapy, has been reported to be effective in improving prognosis, exercise tolerance, and quality of life and reducing the risk of rehospitalization in patients with cardiac disease [3]. However, patients with treatment-resistant severe heart failure may have difficulty performing exercise therapy.

Expand Waon therapy (WT), which in Japanese means soothing warmth, is a repeated sauna therapy that improves cardiac and vascular endothelial function in patients with chronic heart failure [4]. WT is a treatment option for patients with severe heart failure who cannot undergo exercise therapy. It improves the clinical symptoms and prognosis of patients with heart failure [5]. Peripheral vasodilation associated with increased body temperature immediately leads to decreased pre-post load and increased cardiac output and alleviates the clinical symptoms associated with peripheral circulatory disturbances in patients with heart failure. However, WT has low versatility because it requires special equipment.

Lower-leg bathing is a simple thermo-therapeutic approach. According to a study conducted among healthy individuals, lower-leg bathing at 42°C for 20 min can be expected to improve peripheral circulation to the same degree as full-body bathing [6]. Furthermore, lower-leg bathing can be safely performed in patients with acute coronary syndrome in the intensive care unit [7]. Therefore, lower-leg bathing is expected to be a therapeutic option for patients with refractory severe heart failure who have difficulty receiving exercise therapy. However, to our knowledge, lower-leg bathing has not been studied from the perspective of alleviating the symptoms of heart failure. Herein, we report a case of a patient with pulmonary arterial hypertension in which lower-leg bathing enabled the introduction of exercise therapy.

## Case presentation

A woman in her 70s (height: 151 cm, weight: 50.1 kg, and body mass index: 20.9 kg/m^2^) having connective tissue disease-pulmonary arterial hypertension associated with scleroderma was on home oxygen therapy (3 L/min). She experienced shortness of breath during exercise and worsening pulmonary artery hypertension as estimated by transthoracic echocardiography (TTE). Hence, she was admitted for re-evaluation by right heart catheterization (RHC) and medication adjustment.

In addition to scleroderma, she had a history of severe aortic stenosis after transcatheter aortic valve implantation, left ventricular outflow tract obstruction (systolic anterior movement+), postoperative left breast cancer, subclinical hypothyroidism, and postoperative colonic polyps. Her medications included selexipag 1.6 mg, macitentan 10 mg, tadalafil 40 mg, bisoprolol 3.75 mg, spironolactone 25 mg, azosemide 45 mg, aspirin 100 mg, vonoprazan fumarate 20 mg, levothyroxine sodium hydrate 75 µg, dry iron sulfate 210 mg, and butyrate. She had smoked for 10 × 30 years (currently a non-smoker) and did not drink alcohol. Upon admission, her vital signs were as follows: blood pressure (BP), 90/52 mmHg; heart rate, 59 bpm; and sinus rhythm. The laboratory data at admission are shown in Tables 1, 2. The medication doses received by the patient were determined to be the maximum that could be administered. Therefore, cardiac rehabilitation was initiated on day 6 to introduce exercise as a non-pharmacological therapy.

**Table 1:** Laboratory data on admission WBC: White blood cell; RBC: Red blood cell; Hb: Hemoglobin; Hct: Hematocrit; Plt: Platelets; LDH: Lactate dehydrogenase; ALP: Alkaline phosphatase; T. bil: Total bilirubin; AST: Aspartate aminotransferase; ALT: Alanine aminotransferase; γGTP: Gamma-glutamyl transpeptidase; TP: Total protein; Alb: Albumin; CK: Creatine kinase; CK-MB: Creatine kinase-myocardial band; T. Chol: Total cholesterol; LDL: Low-density lipoprotein; HDL: High-density lipoprotein; TG: Total glucose; BUN: Blood urea nitrogen; Cre: Creatinine; UA: Uric acid; CRP: C-reactive protein; NTproBNP: N-terminal prohormone of brain natriuretic peptide; HbA1c: Glycosylated hemoglobin; BS: Blood sugar; LVEF: Left ventricular ejection fraction; LAD: Left atrial dimension; LVDd: Left ventricular diastolic dimension; LVDs: Left ventricular systolic dimension; PR: Pulmonary regurgitation; TR: Tricuspid regurgitation; TRPG: Tricuspid regurgitation pressure gradient; LVOT: Left ventricular outflow tract; VC: Vital capacity; FVC: Forced vital capacity; FEV1.0: Forced expiratory volume in the first second; DLCO: Diffusing capacity of the lung for carbon monoxide; HGS: Handgrip strength; SPPB: Shot physical performance battery; MIP: Maximal inspiratory pressure; MEP: Maximal expiratory pressure; 6MD: Six-minute walk distance; N/A: Not applicable.

Test	Variables	Unit	Results	Reference range	Test	Variables	Unit	Results	Reference range
L/D	WBC	10^3^/μL	8.4	3.3-8.6	L/D	NTproBNP	pg/mL	6175	<125
	RBC	10^4^/μL	456	386-492		HbA1c	%	6.2	4.6-6.2
	Hb	g/dL	12.4	11.6-14.8		BS	mg/dL	90	70-110
	Hct	%	38.3	35.1-44.4	UCG	LVEF	%	73.6	>50
	Plt	10^3^/μL	270	158-348		LAD	mm	50	19-40
	AST	IU/L	20	13-30		LVDd	mm	41.5	39-55
	ALT	IU/L	7	7-23		LVDs	mm	17.5	22-42
	LDH	U/L	228	124-222		PR peak	m/s	3.27	N/A
	ALP	U/L	100	106-322		PR end	m/s	1.98	N/A
	T. bil	mg/dL	0.5	0.4-1.5		TR flow velocity	m/s	4.45	<2.8
	γGTP	IU/L	155	9-32		TRPG	mmHg	81	<40
	TP	g/dL	7.3	6.6-8.1		LVOT peak velocity	m/s	4.15	<30
	Alb	g/dL	3.9	4.1-5.1	PFT	VC	L	1.91	N/A
	CK	U/L	34	41-153			% predicted	81.6	>80
	CK-MB	U/L	<9	<12		FVC	L	1.72	N/A
	T. Chol	mg/dL	177	130-220			% predicted		>70
	TG	mg/dL	142	30-150		FEV1.0	L	1.36	N/A
	HDL	mg/dL	45	40-96			% predicted	79.5	>70
	LDL	mg/dL	101	60-139		DLCO	ml/min/mmHg	4	N/A
	BUN	mg/dL	13.2	8-20			% predicted	28.8	80-120
	Cre	mg/dL	0.68	0.46-0.79	Motor function	HGS	kg	8.3/6.8	>18
	UA	mg/dL	8.1	2.3-7		Gait speed	m/s	0.63	>1.0
	Na	mEq/L	139	138-145		SPPB	point	10	12
	K	mEq/L	4.3	3.6-4.8		MIP	cmH_2_O	38.1	>60
	Cl	mEq/L	107	101-108		MEP	cmH_2_O	52.6	N/A
	CRP	mg/dL	3.1	<0.03		6MD	m	153	>300

**Table 2:** Result of right heart catheterization LLB: Lower-leg bathing; CO: Cardiac output; CI: Cardiac index; mPAWP: Pulmonary artery wedge pressure; mPAP: Mean pulmonary arterial pressure; PVR: Pulmonary vascular resistance.

	Unit	Before LLB	After LLB
Day from admission	day	1	34
CO	L/min	3.31	4.53
CI	L/min/m^2^	2.3	3.24
mPAWP	mmHg	14	11
mPAP	mmHg	68	55
PVR	mmHg	16.31	9.7

At the start of cardiac rehabilitation, the patient could visit the lavatory independently. However, her physical function was impaired (Table 1). In addition, her BP and oxygen saturation (SpO_2_) decreased with mild activities such as standing (supine: BP, 117/58 mmHg; pulse rate (PR), 87 bpm; and SpO_2_, 97%; after standing: BP, 87/54 mmHg; PR, 76 bpm; and SpO_2_, 87%; and after ambulation: BP, 74/45 mmHg; PR, 75 bpm; and SpO_2_, 85%), and shortness of breath was observed. This made it difficult to introduce moderate-to-vigorous exercise therapy for the patient. Therefore, lower-leg bathing was introduced as an alternative to WT. Lower-leg bathing was performed once a day, three times a week at 42°C for 20 min per session using a footbath machine with a heat-retention function (KS-N1010; Nippon Deniken Co., Ltd., Tokyo, Japan).

During each session, vital signs were checked before the lower-leg bath, every 5 min during the lower-leg bath, and after the lower-leg bath. In addition, earlobe blood flow (EBF), an index of peripheral circulation, was measured using a wireless Doppler laser blood flow meter (Pocket LDF®; JMS Co., Ltd., Tokyo, Japan). It was measured continuously during lower-leg bathing, and the rate of change before and after lower-leg bathing was calculated using the value before leg bathing as a baseline value. In addition, to evaluate the circulatory dynamics during movement, circulatory parameters, including EBF, were assessed before and after walking on both the first and last days of cardiac rehabilitation.

**Figure 1:**
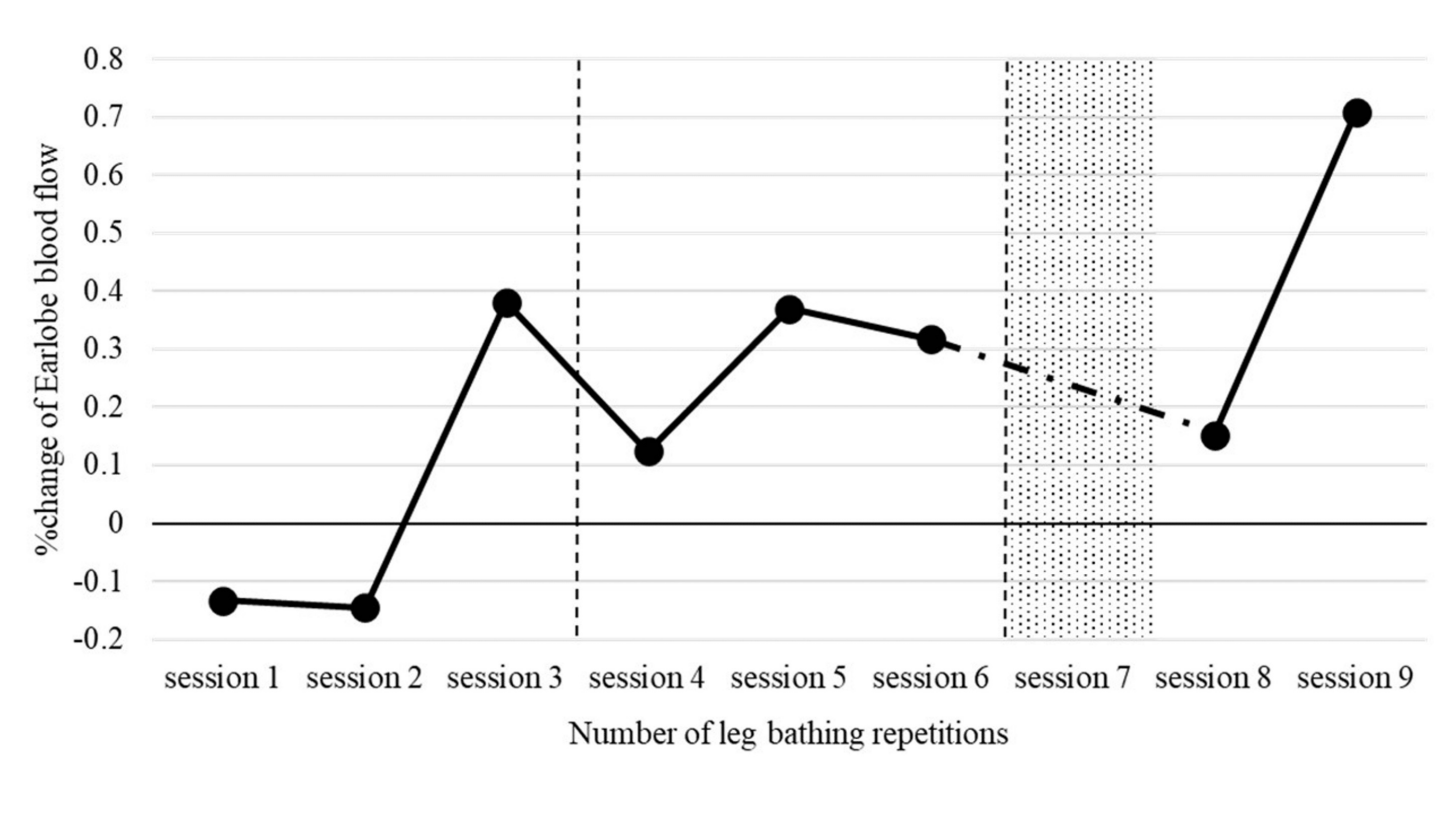
Changes in the rate of change in earlobe blood flow (EBF) before and after lower-leg bathing The patient bathed three times/week for three weeks from day 13 to 33. EBF increased significantly during the ninth session, and subjective changes such as increased sweating, lightening of the legs, and feeling hot enough to remove the jacket were observed.

Lower-leg bathing was performed eight times during three weeks from day 12 to 33. Figure 1 shows the % change in EBF during lower-leg bathing. EBF was low (-13%) during the first lower-leg bathing but increased thereafter. The % EBF increased to 38% during the third lower-leg bathing. At the same time, the patient experienced subjective changes such as mild sweating and feeling warm and seemed comfortable while moving. As such, she was able to perform ambulation practice for approximately 15 min. The seventh session in the third week could not be conducted once because of anemia and was thus conducted twice.

During the ninth (final) session, the % EBF increased to 71%, and subjective changes were observed, such as a significant increase in sweating, lightening of the legs, and the body becoming hot enough to take off the jacket. In addition, BP increased with ambulation (before ambulation: BP, 71/55 mmHg; HR, 58 bpm; SpO_2_, 97%; after ambulation: BP, 84/40 mmHg; PR, 64 bpm; SpO_2_, 93%). RHC on the 34th day showed decreased pulmonary vascular resistance and increased cardiac output (Table 2). Percutaneous transluminal septal myocardial ablation [8] was performed for left ventricular outflow tract obstruction, and muscle strength training with body weight and walking was introduced. The tricuspid regurgitation pressure gradient (TRPG) after admission was worsened to 100.8 mmHg on day 21 (98.4 mmHg on day 28) but recovered to 76.4 mmHg on day 38. The patient was discharged on day 45. After discharge, she continued monthly outpatient cardiac rehabilitation and aerobic exercise on a bicycle ergometer. The six-month follow-up after discharge is shown in Table 3.

**Table 3:** Laboratory data six months after discharge WBC: White blood cell; RBC: Red blood cell; Hb: Hemoglobin; Hct: Hematocrit; Plt: Platelets; TP: Total protein; Alb: Albumin; BUN: Blood urea nitrogen; Cre: Creatinine; UA: Uric acid; CRP: C-reactive protein; NTproBNP: N-terminal prohormone of brain natriuretic peptide; LVEF: Left ventricular ejection fraction; LAD: Left atrial dimension; LVDd: Left ventricular diastolic dimension; LVDs: Left ventricular systolic dimension; PR: Pulmonary regurgitation; TR: Tricuspid regurgitation; TRPG: Tricuspid regurgitation pressure gradient; LVOT: Left ventricular outflow tract; HGS: Handgrip strength; SPPB: Shot physical performance battery.

Test	Variables	Unit	Results	Reference range
L/D	WBC	10^3^/μL	5.1	3.3-8.6
	RBC	10^4^/μL	406	386-492
	Hb	g/dL	12.7	11.6-14.8
	Hct	%	38.7	35.1-44.4
	Plt	10^3^/μL	130	158-348
	TP	g/dL	5.9	6.6-8.1
	Alb	g/dL	3.8	4.1-5.1
	BUN	mg/dL	16.2	8-20
	Cre	mg/dL	0.60	0.46-0.79
	Na	mEq/L	142	138-145
	K	mEq/L	4.2	3.6-4.8
	Cl	mEq/L	112	101-108
	CRP	mg/dL	<0.03	<0.03
	NTproBNP	pg/mL	7378	<125
UCG	LVEF	%	No data	>50
	LAD	mm	50.4	19-40
	LVDd	mm	45.4	39-55
	LVDs	mm	29.7	22-42
	PR peak	m/s	3.73	N/A
	TR flow velocity	m/s	4.4	<2.8
	TRPG	mmHg	77.4	<40
	LVOT peak velocity	m/s	1.48	<30
Motor function	HGS	kg	8.3/6.5	>18
	Gait speed	m/s	0.52	>1.0
	SPPB	Point	7	12

**Figure 2:**
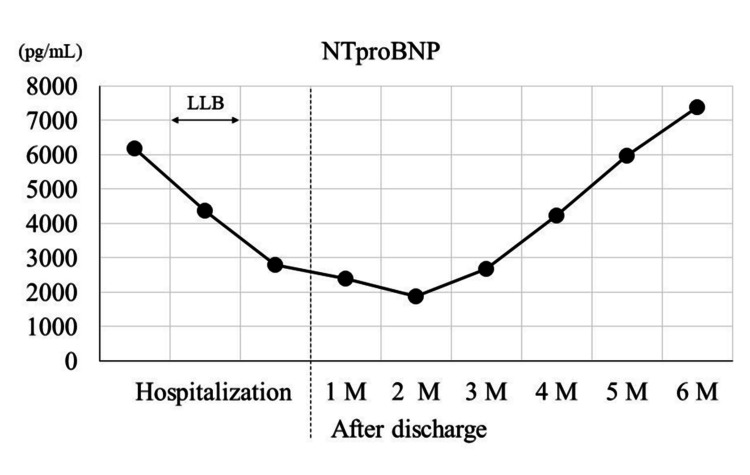
The trend of NTproBNP every month from admission to six months after discharge NTproBNP: N-terminal prohormone of brain natriuretic peptide; LLB: Lower-leg bathing; M: Month.

Figure 2 shows the trend of NT pro-B-type natriuretic peptide (BNP) every month from admission to six months after discharge.

## Discussion

In the present case, repeated lower-leg bathing was provided as an alternative to WT, which alleviated the symptoms of heart failure. The improvement in the peripheral circulation was also confirmed by the monitoring of EBF.

WT is a hyperthermic treatment for heart failure. After exposure to mild heat at 60°C for 30 min in a dry far-infrared sauna, the patient is placed in a supine position outside the sauna and kept warm for 15 min by covering with a blanket [5]. WT alleviates the clinical symptoms associated with peripheral circulatory disturbances in patients with heart failure, as peripheral vasodilation associated with an increased body temperature leads to decreased pre-post load and increased cardiac output [9]. It is also known to reduce preload due to pulmonary vasodilation, mitral regurgitation, and pulmonary artery wedge pressure [10]. In repeat WT, increased shear stress is known to increase peripheral vascular blood flow and vascular endothelial expression of nitric oxide [11]. Patients with alleviated clinical symptoms showed a significant improvement in % flow-mediated vasodilatation (% FMD), whereas patients with unchanged clinical symptoms did not [11]. Thus, WT may improve vascular endothelial function and reduce the clinical manifestations of HF.

Our patient had symptomatic hypotension and hypoxemia and was unable to receive additional medication or exercise therapy. Therefore, we considered WT as a possible treatment option. However, our hospital does not have a dry far-infrared sauna; therefore, we proposed lower-leg bathing as an alternative method. Ghadicolaei et al. reported that a lower-leg bath at 41°C for 20 min for three consecutive days reduced severe sleep disturbance without any adverse events in a patient with acute coronary syndrome in the coronary care unit while evaluating the effect of lower-leg bathing on sleep quality [7]. Yoon et al. reported that in patients with mild-to-moderate coronary artery disease, lower-leg bathing at 40°C for 30 min significantly improved coronary flow reserve without side effects and that this approach can be used with little risk of coronary events if performed appropriately [12]. Thus, lower-leg baths can be safely used to treat cardiovascular diseases. In a preliminary study, we observed a 1.7-fold increase in EBF after a 20-minute lower-leg bath at 42°C [6].

Unlike full-body bathing, lower-leg bathing increases the blood flow in the non-immersed area to dissipate body heat. In other words, an increase in the EBF away from the immersed area indicates an improvement in blood flow throughout the body. We expected a decrease in preload and afterload due to peripheral vasodilatation during lower-leg bathing, as in the case of WT. Although the patient did not respond well to single-lower-leg bathing, repeated lower-leg bathing increased EBF within approximately three weeks, and clinical symptoms and cardiac catheterization results improved during the same period. No additional medication was administered during this period. Improvement in symptoms, especially with exercise, has allowed the introduction of exercise therapy. Although we were unable to directly confirm changes in body temperature or vascular endothelial function, this case suggests that thermotherapy is a non-pharmacological treatment modality for heart failure and that lower-leg bathing, a form of thermotherapy, may be a useful treatment.

This case showed good short-term results in clinical symptoms and PAP, but no long-term effects were observed on echocardiographic results such as TRPG. Asystematic review on the effects of short-term WT also found improvements in BNP and clinical symptoms, but not necessarily in echocardiographic indices [13]. This finding aligns with the report by Kihara et al. [11], and the results of this case are consistent with previous studies. In PH, there are rare cases in which echocardiographic indices do not improve even after pulmonary artery pressure is reduced by treatment, and these cases require careful follow-up and management.

PVR is sensitive to the effects of both blood flow and pressure and may not accurately reflect pulmonary circulation [14,15]. Therefore, careful interpretation of the effect of lower-leg bathing is necessary. WT should be administered twice a week on an outpatient basis to maintain its effectiveness after discharge [16]. NTproBNP remained low until three months after discharge, after which it would increase again, suggesting that long-term continuation of the lower-leg bathing was necessary to maintain the effects. However, her return to daily life may have been affected by changes in activity level and diet. Thus, the impact of lower-leg bathing on the long-term prognosis of this patient is unknown. However, lower-leg bathing was expected to reduce clinical symptoms by improving peripheral circulation [9], and its short-term effect was observed by EBF. Although lower-leg bathing has no long-term effects, it may be a treatment option for severe PH when exercise therapy is difficult to introduce.

## Conclusions

We showed that leg baths are effective for alleviating the symptoms of severe pulmonary arterial hypertension refractory to medication. Three weeks later, the patient’s peripheral circulation improved, symptoms associated with exercise (walking) were alleviated, and exercise therapy could be introduced. However, TRPG and NTproBNP returned to pre-intervention levels six months after discharge. Although lower-leg bathing can temporarily alleviate the patient’s symptoms, it did not show a long carry-over effect. It may be one of the treatment options for severe PH when exercise therapy is difficult to introduce.
